# Estimation of tomato water status with photochemical reflectance index and machine learning: Assessment from proximal sensors and UAV imagery

**DOI:** 10.3389/fpls.2023.1057733

**Published:** 2023-04-06

**Authors:** Zhehan Tang, Yufang Jin, Patrick H. Brown, Meerae Park

**Affiliations:** ^1^ Department of Land, Air and Water Resources, University of California, Davis, Davis, CA, United States; ^2^ Department of Plant Sciences, University of California, Davis, Davis, CA, United States

**Keywords:** photochemical reflectance index, aerial sensing, drone proximal sensors, plant water stress, machine learning, tomatoes

## Abstract

Tracking plant water status is a critical step towards the adaptive precision irrigation management of processing tomatoes, one of the most important specialty crops in California. The photochemical reflectance index (PRI) from proximal sensors and the high-resolution unmanned aerial vehicle (UAV) imagery provide an opportunity to monitor the crop water status efficiently. Based on data from an experimental tomato field with intensive aerial and plant-based measurements, we developed random forest machine learning regression models to estimate tomato stem water potential (*ψ*_stem_), (using observations from proximal sensors and 12-band UAV imagery, respectively, along with weather data. The proximal sensor-based model estimation agreed well with the plant *ψ*_stem_ with *R* ^2^ of 0.74 and mean absolute error (MAE) of 0.63 bars. The model included PRI, normalized difference vegetation index, vapor pressure deficit, and air temperature and tracked well with the seasonal dynamics of *ψ*_stem_ across different plots. A separate model, built with multiple vegetation indices (VIs) from UAV imagery and weather variables, had an *R* ^2^ of 0.81 and MAE of 0.67 bars. The plant-level *ψ*_stem_ maps generated from UAV imagery closely represented the water status differences of plots under different irrigation treatments and also tracked well the temporal change among flights. PRI was found to be the most important VI in both the proximal sensor- and the UAV-based models, providing critical information on tomato plant water status. This study demonstrated that machine learning models can accurately estimate the water status by integrating PRI, other VIs, and weather data, and thus facilitate data-driven irrigation management for processing tomatoes.

## 1 Introduction

Declining water availability and increasing water demand threaten the agricultural sustainability in many arid and semi-arid regions, such as California’s Central Valley (Faunt et al., 2016). Growers have to improve water use efficiency to meet the strict regulation of groundwater usage in the state (Leahy, 2016) and the increasing water price (Lund et al., 2018) in California. This is particularly crucial for processing tomatoes (*Solanum lycopersicum*), one of the leading high-value agriculture commodities in California. With a total area of over 92,200 ha, California produced 11.19 million tons of processing tomatoes in 2019, accounting for more than 90% of the US total production (CDFA, 2020). California’s Mediterranean climate features a highly suitable warm and dry growing season ideal for irrigated tomato production. About 90% of tomato fields in California are irrigated with furrow, sprinkler, and drip irrigation, with a water usage of 7,314 m^3^ per hectare (Cody and Johnson, 2015). Data-driven precision irrigation practices have become increasingly important for agriculture management amid changing climate and water shortage (Abioye et al., 2020). Information on plant water status levels can provide growers with guidance about when to irrigate at the sub-field scale (Bastiaanssen et al., 2000).

Accurate monitoring of plant water status is a crucial step toward precision irrigation management. Pressure chambers are the most accurate and direct measurement of plant water status and are used by a subset of growers to measure the stem water potential (*ψ*_stem_) in the field (Duniway, 1971; Turner, 1988). However, *ψ*_stem_ measurements are labor intensive and are usually only conducted on a small number of plants on infrequent sample dates. Thus, this method is not useful to map the variability of crop water status within the field nor to track the temporal change of water status. Proximal sensing and remote sensing techniques, however, provide an efficient way to monitor plant status across space and time. Relatively cheap proximal sensors mounted on stands, typically including high-frequency-point spectral measurements in the visible, near infrared, and thermal region, have been used to estimate the water status of cotton (Mccarthy et al., 2010), soybean (O’Shaughnessy et al., 2011), and nitrogen status of spring wheat and corn (Tremblay et al., 2009). Proximal observations have also been used to detect soybean foliage disease symptoms (Herrmann et al., 2018) and winter wheat head blight disease (Dammer et al., 2011).

Recently, the advancement in unmanned aerial vehicle (UAV) platforms and miniature sensor technology, coupled with the development of image processing software, has enabled high-resolution crop imaging (Tsouros et al., 2019). Using vegetation indices (VIs) related with canopy structure, chlorophyll content, xanthophyll cycle activity, and sun-induced fluorescence, UAV systems can provide multispectral and hyperspectral imagery and have been successfully applied to weed mapping (Peña et al., 2013; Huang et al., 2018), water stress detection (Suárez et al., 2008; Berni et al., 2009; Baluja et al., 2012; Zarco-Tejada et al., 2013), and nitrogen status monitoring (Hunt et al., 2018; Cai et al., 2019) and have been used for high-throughput phenotyping (Yang et al., 2017; Xie and Yang, 2020). By making use of the difference between foliage and air temperature (Jackson et al., 1981), UAV-based thermal cameras were successfully used for crop water stress monitoring (Zhang et al., 2019), plant disease detection (Calderón et al., 2015), and phenotyping (Gómez-Candón et al., 2016; Sagan et al., 2019; De Swaef et al., 2021).

The photochemical reflectance index (PRI) derived from the decreased reflectance at 531 nm during the de-epoxidation cycle of xanthophyll (Gamon et al., 1997) has shown potential for plant stress detection at the early growth stages (Meroni et al., 2008). PRI values generated from airborne hyperspectral cameras have been used to estimate the *ψ*_stem_ of olive trees with consistent relationships (*R* ^2^ = 0.7, *n* = 10), and the performance of PRI was more consistent than that of normalized difference vegetation index (NDVI) and transformed chlorophyll absorption in reflectance index/optimized soil-adjusted vegetation index (TCARI/OSAVI) (Suárez et al., 2008). Similarly, PRI imagery from UAV-based hyperspectral and multispectral cameras was found to be useful to detect olive verticillium wilt disease (Calderón et al., 2015) and the water status of five different tree species (Ballester et al., 2018). However, the relationship between plant stress levels and PRI can be unstable, as the PRI signal is easily confounded by external or canopy structural effects such as leaf area index (LAI), shadow fraction, vegetation cover, and sun and view zenith angles (Barton and North, 2001; Sims et al., 2011; Moreno et al., 2012; Soudani et al., 2014; Wu et al., 2015). To resolve these problems, studies have been conducted to combine PRI with other vegetation indices. Zarco-Tejada et al. (2013) proposed an index that normalized PRI with structural index renormalized difference vegetation index (RDVI) and chlorophyll index R700/R670. This normalized PRI index outperformed the standard PRI and other VIs in estimating the crop water status (*R* ^2^ = 0.77 *vs*. *R* ^2^ = 0.49).

While most existing studies only explored the relationship between individual vegetation indices and water status indicators, combining information from multiple vegetation indices with machine learning models may provide a more accurate estimation of plant water status (Hassan-Esfahani et al., 2015; Poblete et al., 2017; Loggenberg, 2018; Romero et al., 2018; Das et al., 2021; Tang et al., 2022). In addition, physiological studies have shown that weather conditions, including air temperature, and vapor pressure deficit (VPD) regulate plant stomatal closure and water exchange with the atmosphere and thus directly affect the plant water status (Garnier and Berger, 1985; Ferreira and Katerji, 1992; Ortuño et al., 2006). Weather conditions have not been incorporated with proximal and remote sensing multispectral data for plant water status monitoring in most studies (Das et al., 2021; Tang et al., 2022).

This study aims to develop a robust approach for monitoring the temporal and spatial variability of *ψ*_stem_ in a processing tomato field by utilizing proximal sensing data and UAV multispectral imagery. The specific objectives of this study include the following: (1) to evaluate the plant water status monitoring capability of individual vegetation indices obtained from proximal sensors and UAV imagery, (2) to develop a machine learning model to integrate proximal sensing data and weather data to monitor the temporal change of plant water status throughout the growing season, and (3) to develop a machine learning model to combine UAV imagery and weather data for the accurate mapping of plant water status.

## 2 Materials and methods

### 2.1 Study site and experiment design

**Figure 1:**
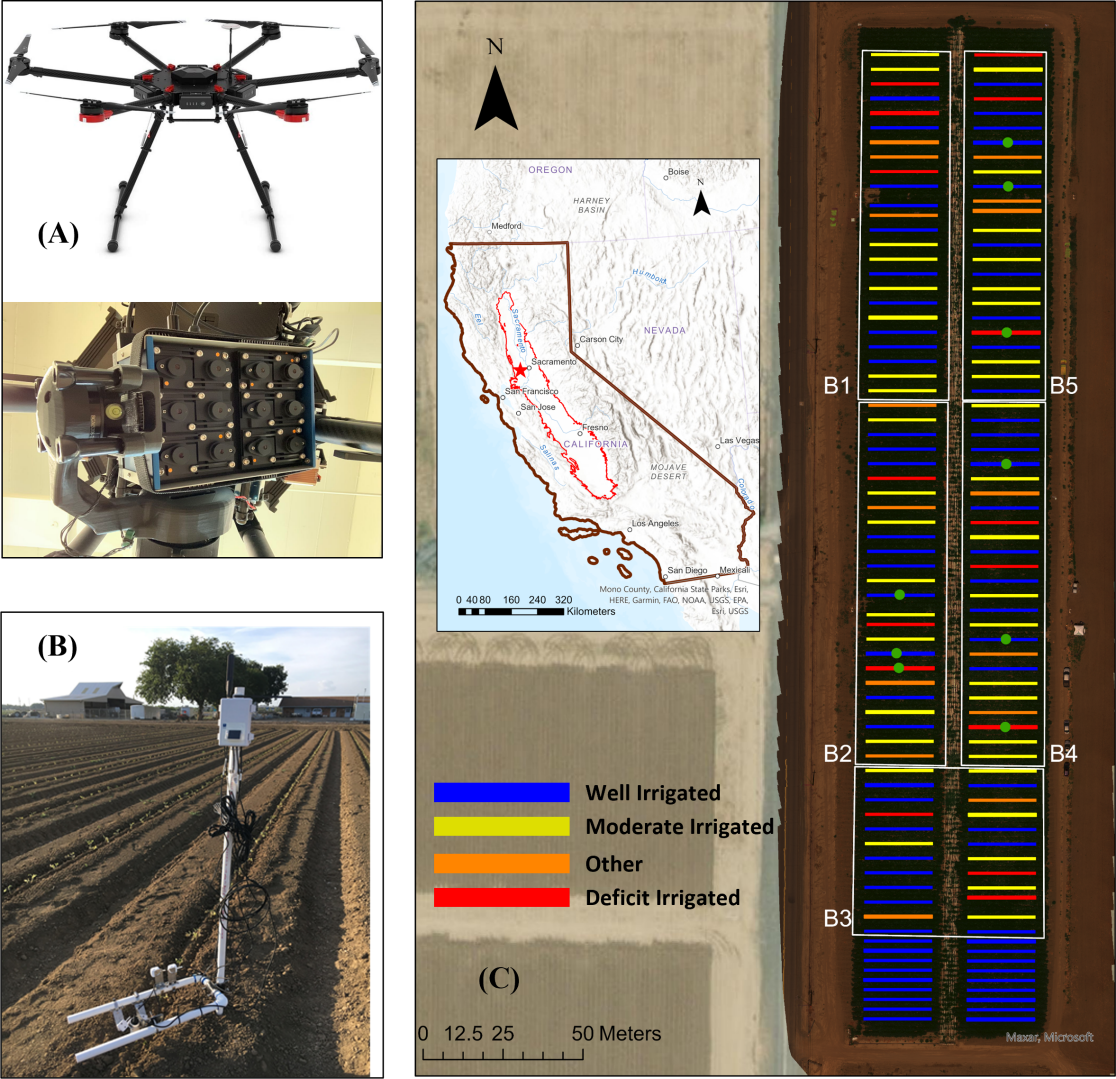
Study area (inset) and experiment design of the processing tomato field in Davis, California. **(A)** Unmanned aerial vehicle (UAV) and multispectral camera used in this study. **(B)** Stands with proximal sensors installed in the field. **(C)** The five blocks (B1, B2, B3, B4, and B5) of the field and the irrigation treatments of different plots. The block boundaries are shown as white polygons on top of the true color composite UAV imagery acquired on June 26, 2019. The blue, yellow, and red colors represent well, moderate, deficit and other irrigation treatments, where 100%, 70%, and 30% of actual evapotranspiration were applied to the plots between reaching full canopy (July 11, 2019) and irrigation cutoff (Aug 26, 2019), respectively. The locations of proximal sensors are shown as green dots.

The study focused on a 1.92-ha processing tomato field in Sacramento Valley near Davis, California (38.54° N, 122.77° W), which features a typical Mediterranean climate with a warm and dry growing season in spring and summer. This site has coarse loamy sand and silt soils on a very flat topography. The field was irrigated through a subsurface drip irrigation system based on the actual evapotranspiration (ET_A_) measured by in-field surface renewal sensors (Tule Technologies, San Francisco, CA, USA). We divided the experimental field into five large blocks, with 720 plots of 30.5 m × 1.5 m arranged in a randomized complete block design (**Figure 1**). The processed tomatoes, *Heinz Variety 1662*, were transplanted on May 2, 2019. In total, 100 plants were planted in each plot.

Three irrigation treatments were implemented in 2019 (**Figure 1**). All plots were fully watered from June 1, 2019 to July 7, 2019 when the plants reached full canopy cover. Afterwards, three types of irrigation treatments were applied to the randomized plots, with applied water equivalent to 35% (deficit irrigated) and 70% (moderately irrigated), and 100% (well irrigated) of ET. Irrigation was cut off for all plots on August 26, 2019 for harvest preparation. The accumulated ET and precipitation were 455 and 1 mm, respectively, during this period (June 1–Aug 26). The accumulated irrigation depths were 363, 394, and 543 mm for deficit-irrigated, moderately irrigated, and well irrigated treatments. All plots were fertilized with 28 kg N/ha before transplant and 174 kg N/ha during the growing season.

### 2.2 Field measurements

Midday stem water potential (SWP, *ψ*_stem)_ was measured with a pressure chamber (PMS Instrument Company, Albany, OR, USA) during 13:00 to 16:00, approximately every 2 weeks from late May to late July and approximately every week from late July to mid-August (**Table 1**). For each block, we selected a minimum of three sample plots for field measurements to represent well-irrigated plots (hereafter referred to as WI; *n* = 5), moderately irrigated plots (MI; *n* = 5), and most severely deficit-irrigated plots (DI; *n* = 5), in addition to other irrigation treatments. The row numbers in the field and center locations were recorded for each sample plot. Six to eight plants were randomly chosen for each sample plot; a mature sunlit leaflet was selected from each sampled plant to be enclosed in a reflective envelope for 3 h to achieve equilibrium with the water potential of the stem. The leaflets were then excised and placed into the pressure chamber for the *ψ*_stem_ measurement (Shackel et al., 1997). For each plot, measurements from all sampled leaves were averaged to represent the mean water potential.

**Table 1:** Information on the ground measurements of stem water potential (*ψ*_stem_) and 12-band aerial imagery acquisition with unmanned aerial vehicle (UAV) over the processing tomato field.

Date	Number of plots with *ψ*_stem_ measurements	UAV flights	Air temperature (°C)	VPD (kPa)
05/31	15	×	27.7	2.34
06/12	15	×	34.1	3.67
06/26	15	✓	26.3	2.11
07/17	15	×	29.4	2.32
07/25	15	×	30.4	3.00
08/01	20	✓	29.5	2.39
08/08	20	×	26.6	1.83
08/14	25	✓	35.9	4.42

Also shown are the corresponding air temperature and vapor pressure deficit (VPD) at noon from the California Irrigation Management Information System weather station nearby.

We obtained the hourly weather data, including air temperature, relative humidity, and solar radiation, from the California Irrigation Management Information System program (https://cimis.water.ca.gov/). The closest station (Davis #6) was located 0.5 km from the experiment field. Hourly data was averaged during 13:00–16:00 to represent mean weather conditions corresponding to field measurements. VPD was also calculated from the noontime average air temperature and relative humidity.

### 2.3 Canopy reflectance measurements from proximal sensors

Three sets of Spectral Reflectance Sensors (METER Group, Pullman, WA, USA) were installed in each of the three blocks (B2, B4, and B5), specifically targeting three WI plots, three MI plots, and two DI plots (**Figure 1**). SRS has been used in many studies for phenotyping and stress detection over agricultural fields (Bai et al., 2016; Magney et al., 2016; Pérez-ruiz et al., 2020; Saiz-Rubio et al., 2021) and measuring the light use efficiency and tracking the pigment change of forests (Gamon et al., 2015; Castro and Sanchez-Azofeifa, 2018; Eitel et al., 2019). We installed the SRS sensors 1.1 m above the tomato canopy on fixed stands after transplanting. They continuously measured the incident radiation with the upward-looking hemispherical sensors (180° field-of-view) and the reflected radiation with the downward-looking field-stop sensors (36° field-of-view) every 5 min. There were three tomato plants within the field of view. Each SRS sensor has four spectral channels centered at 532, 570, 650, and 810 nm, each with a 10-nm full-width half-maximum band width. The readings from the paired up- and downward-looking sensors were combined to calculate the reflectance for each wavelength first and then PRI and NDVI using the following equations:


$$\text{PRI}=\left(\rho_{532}-\rho_{570}\right)/\left(\rho_{532}+\rho_{570}\right)$$

$$\text{NDVI}=\left(\rho_{810}-\rho_{650}\right)/\left(\rho_{810}+\rho_{650}\right)$$

For each sensor, the continuous NDVI and PRI data was averaged using the moving window of every five readings to reduce the high frequency noise. The NDVI and PRI values during 12:00–14:00 were averaged to represent the noon time values, respectively, and to minimize the directional impacts of changing the sun angle at different times of the day (Magney et al., 2016). These consistent daily time series of NDVI and PRI around noon were then used for further analysis. Data from the sensors in the deficit-irrigated plot in block 5 was excluded in this study due to the abnormal sensor performance during the mid-season.

### 2.4 UAV imagery acquisition and processing

The UAV imaging system consists of a Macaw-12 camera (Tetracam, Chatsworth, CA, USA), a FirePoint™ GPS unit, and an incoming light sensor fully integrated on a DJI Matrice 600 Pro hexacopter (DJI, Shenzhen, China). The multispectral camera records reflected solar radiation at 12 customized spectral bands centered at 450, 480, 531, 550, 570, 670, 700, 720, 740, 800, 900, and 970 nm, with 10–20 nm full-width at half-maximum. The light sensor was installed on the top of the hexacopter to measure the incoming solar radiation for each of the corresponding bands.

We collected multispectral aerial images on June 26 and August 1 and 14, 2019. All flights were conducted around solar noon to reduce the impacts of shadows and minimize the variation in light condition. The same flight plan was designed and executed with 85% front overlap and 85% side overlap. The UAV system was flown at 50 m above ground level, resulting in aerial images at 2.5-cm resolution. For geo-reference purposes, we placed six highly visible aluminum flashing targets across the field as ground control points (GCPs) and recorded their GPS coordinates using Trimble Geo 7x (Trimble, Westminster, CO, USA).

The raw multispectral images were first imported in PixelWrench software (Tetracam, Chatworth, CA, USA) to calculate reflectance from radiance using the smoothed incoming light sensor data and to align images from different bands. The preprocessed reflectance images were then stitched together with Agisoft Metashape Pro (Agisoft, St. Petersburg, Russia). The software generated automatic tie points initially and used the coordinates of the GCPs to ensure their geolocation accuracy. The point clouds, 3D textured mesh, and digital surface model were subsequently created based on the automatic tie points. The orthomosaics of reflectance maps were finally generated at 2.5-cm resolution for each spectral band.

Multiple widely used VIs were further calculated using the reflectance from the orthomosaics (**Table 2**). We included the indices related to canopy structure—*e*.*g*., NDVI, RDVI, and enhanced vegetation index (Roujean and Breon, 1995; Jiang et al., 2008), indices based on light absorption by chlorophyll content—*e*.*g*., TCARI, TCARI/OSAVI, red edge ratio (RER), and normalized difference red edge (NDRE) (Vogelmann et al., 1993; Barnes et al., 2000; Haboudane et al., 2002), and the VIs based on xanthophyll cycle activity such as PRI and PRI_550_ (Gamon et al., 1992) and the combined index that normalized PRI with structure and chlorophyll index (Zarco-Tejada et al., 2013).

**Table 2:** The vegetation indices (VIs) calculated in this study, and the coefficients of correlation (r) between each VI derived from unmanned aerial vehicle imagery and measured stem water potential (*ψ*_stem_) for each individual flight and for all data.

Index	Equation	June 26 (*n* = 15)	August 01 (*n* = 20)	August 14 (*n* = 25)	3 (*n* = 60)
Structural index					
NDVI	$\frac{R_{800}-R_{670}}{R_{800}+R_{670}}$	61**	0.63***	0.76***	0.74***
RDVI (Roujean and Breon, 1995)	$\frac{R_{800}-R_{670}}{{\left(R_{800}+R_{670}\right)}^{0.5}\;}$	42	0.58**	0.73***	0.57***
EVI (Jiang et al., 2008)	$2.5\times \frac{R_{800}-R_{670}}{R_{800}+6\times R_{670}-7.5\times R_{450}+1\;}$	41	0.57***	0.73***	0.57***
Chlorophyll index					
TCARI (Haboudane et al., 2002)	$3\left[\left(R_{700}-R_{670}\right)-0.2\left(R_{700}-R_{550}\right)\left(R_{700}/R_{670}\right)\right]$	40	0.37	0.15	0.73***
RER1 (Vogelmann et al., 1993)	$R_{700}/R_{670}$	49*	0.77***	0.71***	0.40***
RER2 (Vogelmann et al., 1993)	$R_{720}/R_{670}$	56**	0.69***	0.78***	0.80***
TCARI/OSAVI (Haboudane et al., 2002)	$\frac{TCARI}{\left(1+0.16\right)*\left(R_{800}-R_{670}\right)/\left(R_{800}+R_{670}+0.16\right)}$	18	0.00	-0.29	-0.76***
NDRE1 (Barnes et al., 2000)	$\frac{R_{800}-R_{700}}{R_{800}+R_{700}}$	53**	0.51**	0.71***	0.75***
NDRE2 (Barnes et al., 2000)	$\frac{R_{800}-R_{720}}{R_{800}+R_{720}}$	39	0.44**	0.61***	0.31**
Xanthophyll index					
PRI (Gamon et al., 1997)	$\frac{R_{531}-R_{570}}{R_{531}+R_{570}}$	32	0.22	0.71***	0.84***
PRI550 (Gamon et al., 1992)	$\frac{R_{531}-R_{550}}{R_{531}+R_{550}}$	10	-0.55**	-0.15	0.10
Combined index					
PRInorm1 (Zarco-Tejada et al., 2013)	PRI/(NDVI*RER1)	47*	0.40*	0.75***	0.84***
PRInorm2 (Zarco-Tejada et al., 2013)	PRI/(NDVI*RER2)	53**	0.44*	0.78***	0.86***
PRInorm3 (Zarco-Tejada et al., 2013)	PRI/(RDVI*RER1)	54**	0.44*	0.76***	0.79***
PRInorm4 (Zarco-Tejada et al., 2013)	PRI/(RDVI*RER2)	59**	0.46**	0.78***	0.82***

The significance levels are indicated by *p ≤ 0.1, **p ≤ 0.05, and ***p ≤ 0.01.

**Figure 2:**
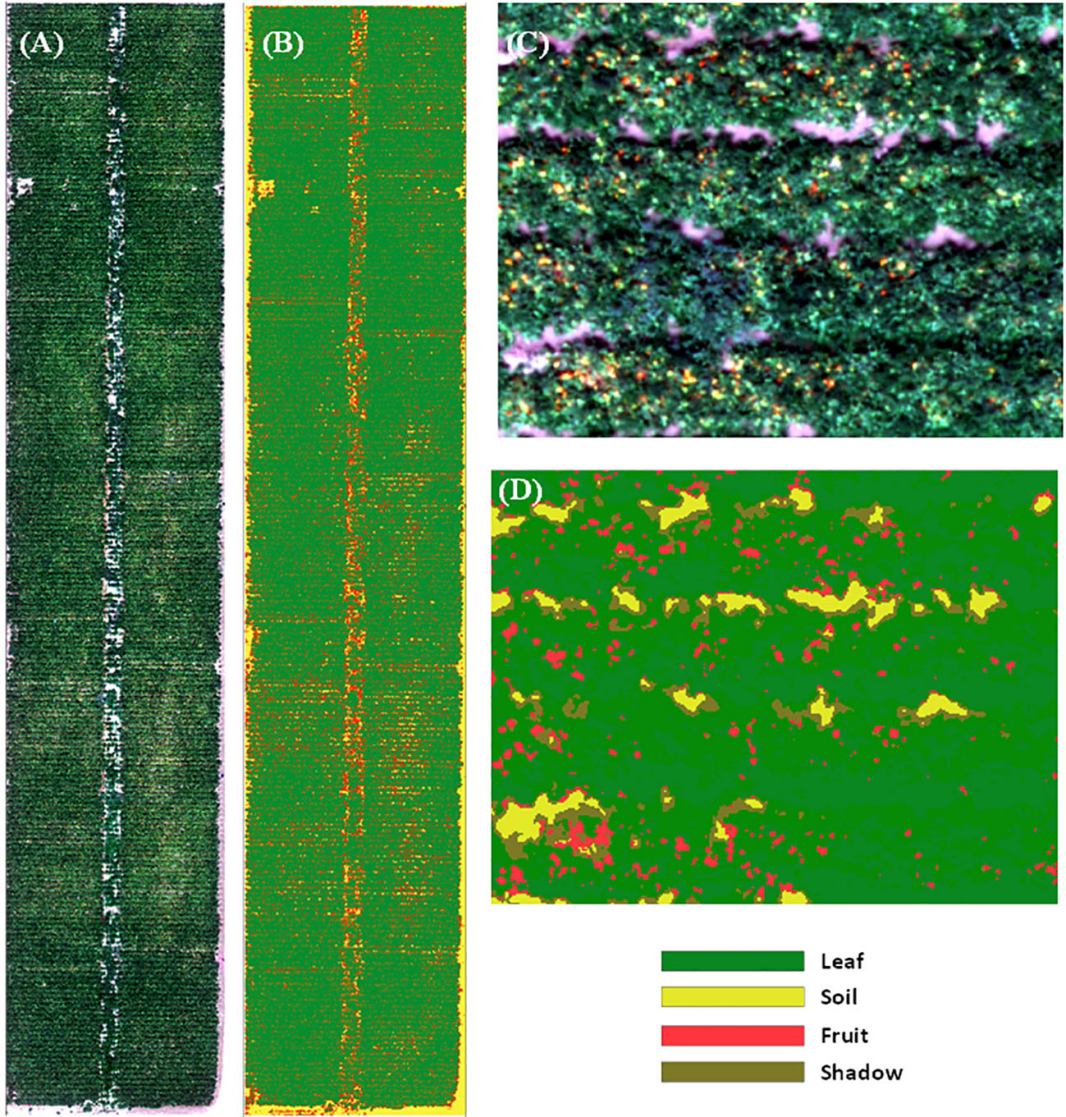
RGB imagery of the entire experiment field **(A)** and a subset of the field **(C)**. The supervised classification results of the entire field **(B)** and a subset of the field **(D)** based on the unmanned aerial vehicle imagery on August 10, 2019. The imagery was classified into four categories (leaf, fruit, soil, and shadow).

To separate the tomato leaves from tomato fruits and soil background, we classified the processed UAV imagery into four categories (leaf, fruit, soil, and shadow) using a supervised machine learning approach. We used the support vector machine approach in ArcGIS Pro (ESRI, Redlands, CA, USA). This method aims to find the best hyperplane in the multi-spectral feature space that optimally separates classes. It has been widely used for land cover classification based on satellite (Huang et al., 2002) and UAV images (Ma et al., 2017). For each class, we randomly selected 20 regions of interest through visual interpretation, and all pixels within these polygons were then used for training. The pixels identified as fruits, soil, and shadow from the classified map (**Figure 2**) were then masked out, and only the remaining vegetated pixels were used for further analysis.

### 2.5 Statistical analysis

We analyzed the statistical relationships between ground-measured *ψ*_stem_ and the VIs derived from the proximal sensors and UAV imagery, respectively. Univariable linear correlation was performed first to explore the potential of estimating *ψ*_stem_ with remotely sensed observations. To remove potential uncertainties in field measurements and location matching errors, the analysis was done at the individual plot level. Specifically, for each sample plot, measured *ψ*_stem_ was averaged over all sampled plants, representing the mean water status condition. A single-day analysis was first performed with proximal sensor data for each of the 8 days when ground measurements were available, with plot samples ranging from 15 to 25. A separate analysis was then done by pooling the proximal data for three dates before July 7 when the canopy reached full cover (*n* = 8 × 3) and for five dates afterwards (*n* = 8 × 5).

Similarly, correlations between ground-measured *ψ*_stem_ and UAV-based VIs were analyzed for 3 days where UAV flights were conducted. For each flight, we delineated the boundary for each sample plot where field measurements of *ψ*_stem_ were conducted based on the UAV imagery and the location of the plot. The values of spectral reflectance and VIs were extracted and averaged over all vegetated pixels as described in Section 3.4.

### 2.6 Water status estimation with remote sensing methods

We built machine learning models to estimate the midday *ψ*_stem_ of tomato plants based on available remote sensing data, *i*.*e*., from point-based proximal or UAV imagery-based spectral observations, respectively, considering the complex relationships between remote sensing observations and water status. In particular, the random forest regression (RF) algorithm (Breiman, 2001) was used for this study to combine remote sensing metrics with weather data. Other machine learning approaches, including support vector regression (Smola and Schölkopf, 2004) and extreme gradient boosting regression (Chen and Guestrin, 2016), were also evaluated but had lower performance than the RF models. Therefore, only RF model results were included in the following sections. As an ensemble learning method, RF produces multiple independent decision trees, fits them to random subsets of the training set, and yields optimal results by combining the predictions of the decision trees (Belgiu and Drăgut, 2016). We used the “Caret” package in R for the machine learning model development (Kuhn, 2008).

We trained and cross-validated separate RF models to estimate SWP with observations from the proximal sensors and UAV imagery, respectively. For the proximal sensor-based model, besides PRI and NDVI, air temperature and VPD were also included as predictors to track *ψ*_stem_ throughout the season. For the UAV-based model, we used the 12-band imagery from UAV and included additional VIs for mapping *ψ*_stem_ (**Table 2**). To reduce the number of predictors, the recursive feature elimination (RFE) method was applied, to reduce overfitting and improve model performance. The RFE method iteratively proceeds with the following steps for each specific model: fit the data with all variables using the model, compute the variable importance and discard the variable that has the least importance, and refit the model with the remaining variables. These iterative steps are performed until the model with the least RMSE is found. The remaining variables after feature selection with the RFE method were used for further steps.

The repeated fourfold cross-validation was performed for model development and evaluation. For each of the four folds, 75% of the dataset were randomly selected as the training set, and the remaining 25% were used for validation. The model performance was evaluated using the following statistical metrics for each fold: coefficient of determination (*R* ^2^), root mean square error (RMSE), and mean absolute error (MAE). This process was repeated 10 times. The mean values and standard deviation of the statistical metrics were calculated from the repeated fourfold cross-validation and reported as model performance summary.

### 2.7 Variable importance and response functions

To further understand how each predictor affects the *ψ*_stem_ estimation, we generated the variable importance plots and partial dependence plots from the RF machine learning models. The variable importance is quantified by the increase in the mean sum of squares when an individual variable is excluded from the full model (Strobl et al., 2008) and thus represents each variable’s contribution to the improvement of the model. The partial dependence plots show the relationship between a variable and the predicted outcome of the model by marginalizing over the values of other variables in the model (Lemmens and Croux, 2006).

### 2.8 Stem water potential tracking and mapping

We estimated daily *ψ*_stem_ during the whole growing season over eight sample plots instrumented with the proximal sensors by applying the proximal sensor-based model to the daily time series of PRI and NDVI from proximal sensors, along with weather data. The UAV-based model was also used to produce the *ψ*_stem_ at the pixel level and averaged to the individual plant level over the entire field for 3 days with UAV flight. We further aggregated the predicted *ψ*_stem_ for plants under different treatments and different blocks across 3 days to examine if the predicted *ψ*_stem_ matched the observed spatial and temporal patterns of water status.

## 3 Results

### 3.1 Variations of proximal sensor observations and measured stem water potential

**Figure 3:**
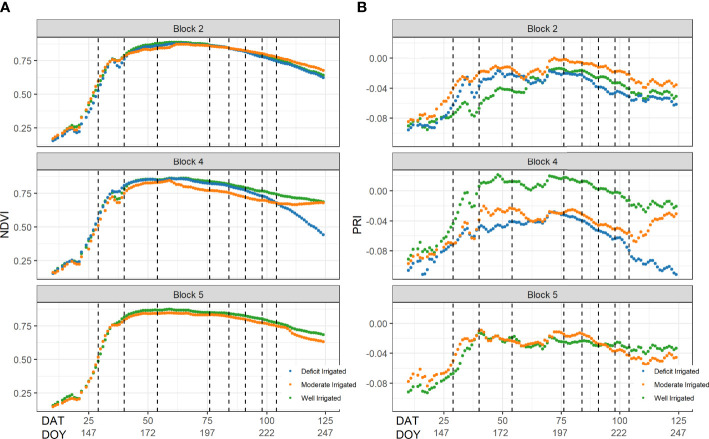
The seasonal changes of **(A)** normalized difference vegetation index (NDVI) and **(B)** photochemical reflectance index (PRI) from the proximal sensors across different plots. The x-axis represents the day after transplanting (DAT) and Julian day of year. The vertical lines represent the dates when the stem water potential was measured on the ground.

NDVI from proximal sensors increased rapidly from below 0.25 in early May to 0.75 in June 12 and slowly reached the plateau in early July (**Figure 3**). This agreed well with the field observations that the tomato plants started to grow fast after being transplanted on May 2 and reached the maximum canopy cover by July 11. Afterwards, NDVI decreased slowly during fruit growth and plant senescence. No significant differences in NDVI were found among eight sample plots during the early growing season, but the deficit-irrigated plots had lower NDVI than the other plots toward the end of the growing season.

**Figure 4:**
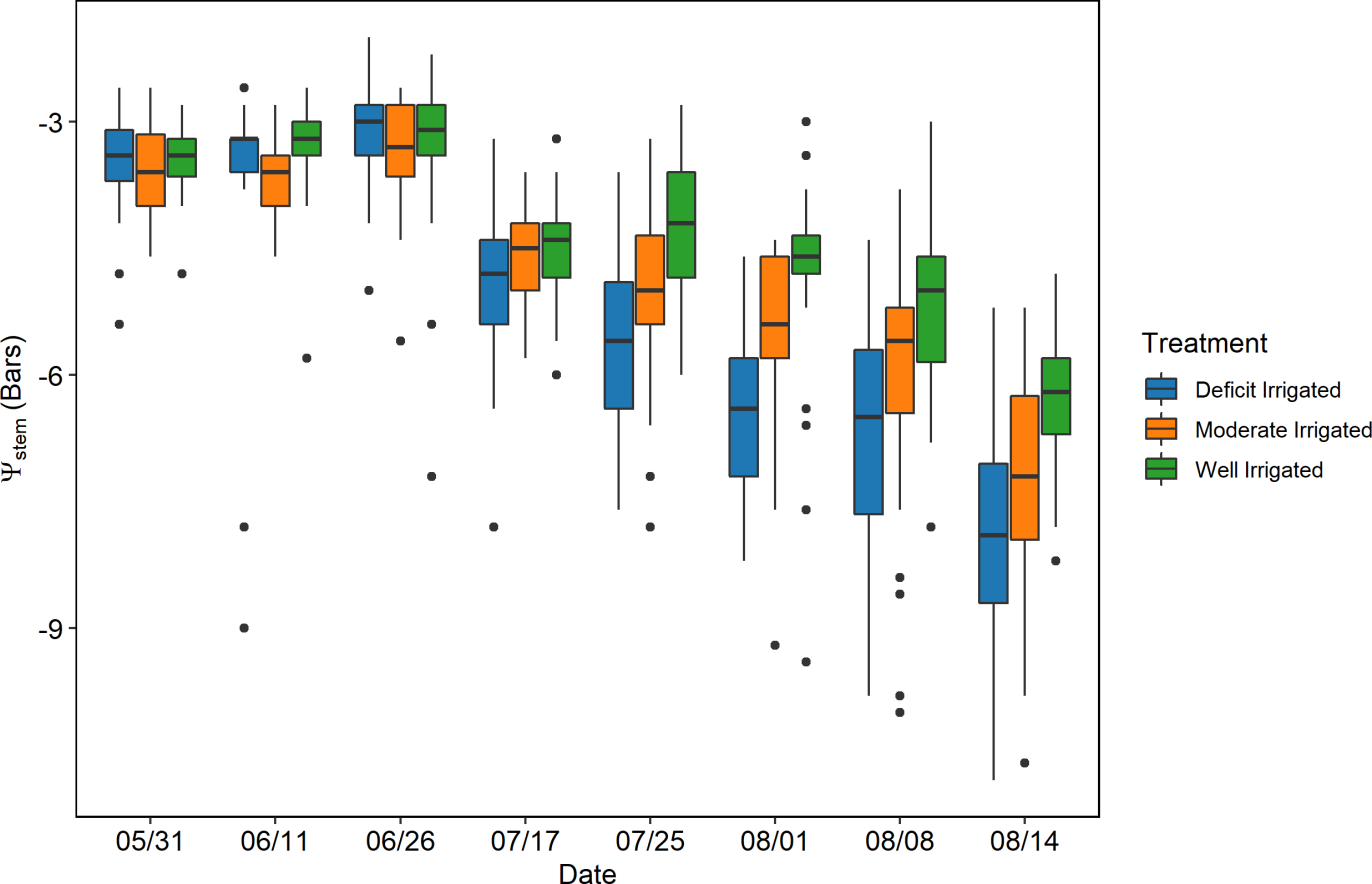
The seasonal change of the stem water potential (*ψ*_stem)_ in the plots where the proximal sensors were located. The red frames represent the dates when the unmanned aerial vehicles imageries were acquired.

The midday stem water potential of processing tomatoes measured during May 31–August in 2019 showed a clear seasonality (**Figure 4**). The plant *ψ*_stem_ values were similar among different plots on the first three sampling days in May and June, with the mean at approximately -3.4 bars. The average *ψ*_stem_ of all sample plants decreased rapidly from -5.0 to -7.4 bars from July 17 to August 14 after the plants reached full canopy and deficit irrigation treatments were applied in this period. A difference in *ψ*_stem_ was found across different deficit irrigation treatments after June 26 and became increasingly significant at the end of growing season (*p*< 0.05) based on the analysis of variance (ANOVA)—for example, the deficit-irrigated plants had lower *ψ*_stem_ than moderately irrigated plants on July 17, but the difference was not significant (*p* > 0.05); however, the *ψ*_stem_ of deficit-irrigated treatment became significantly lower than the other treatments after July 26 (*p*< 0.05) by 0.64–1.22 bars, while moderately irrigated plants had the highest *ψ*_stem._

Similar to NDVI, the PRI values in the eight sample plots increased rapidly from -0.08 before June 12 and then fluctuated approximately -0.02 between June 26 and July 17 (**Figure 3**). Thereafter, the temporal trajectory of PRI followed similar dynamics of the ground measured *ψ*_stem,_ decreasing from -0.04 on July 17 to -0.06 on August 14. The PRI values of plants under deficit-irrigated treatments were approximately 0.01 lower than those of plants under the other treatments, after July 17, in both block 2 and block 4.

### 3.2 Estimating and tracking water status with proximal sensor observations

**Figure 5:**
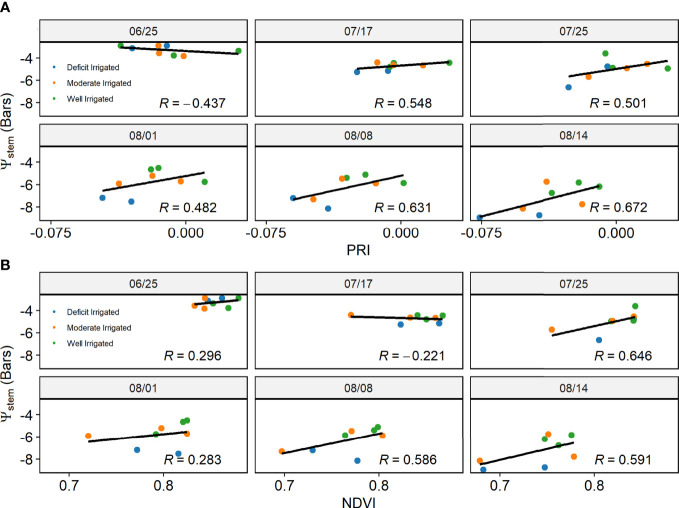
The correlation between **(A)** photochemical reflectance index and **(B)** normalized difference vegetation index from proximal sensors and ground measured stem water potential (*ψ*_stem_) for each individual day.

**Figure 6:**
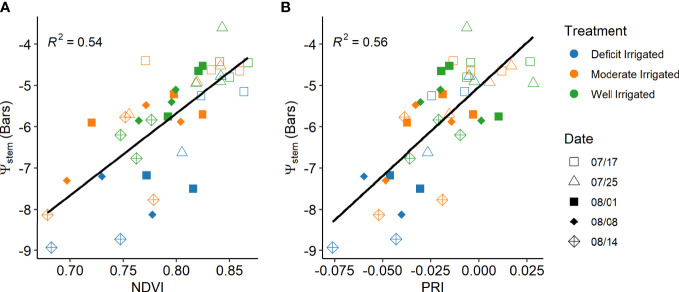
Relationships of the mid-day stem water potential (*ψ*_stem_) with concurrent vegetation indices from proximal sensors: **(A)** normalized difference vegetation index and **(B)** photochemical reflectance index after the tomato plants reached maximal canopy size and different irrigation treatments were applied.

Over each individual day after July 17, the PRI from proximal sensors was highly correlated with the measured *ψ*_stem_ across individual plants, with correlation coefficients (*r*) greater than 0.48. However, the correlation was lower before reaching the maximal canopy cover, *e*.*g*., *r* = -0.44 on June 26 (**Figure 5**). When pooling data together from all days after July 17, we found a significant relationship between the ground measured *ψ*_stem_ and the proximal sensor-measured PRI (*R* ^2^ = 0.56, *p*< 0.001) (**Figure 6A**). This relationship was not significant for the sample days before full canopy cover (*R* ^2^ = 0.01, *p* > 0.1). NDVI and *ψ*_stem_ had low correlation on June 26 and July 17 (|*r*|< 0.3, *p* > 0.1) and had a higher correlation on July 25 and August 8 and 14 (*r* > 0.58, *p*< 0.1). Over all 5 days since July 17, there was a significant relationship between the NDVI from proximal sensors and ground-measured *ψ*_stem_ (*R* ^2^ = 0.554, *p*< 0.001) (**Figure 6B**).

**Figure 7:**
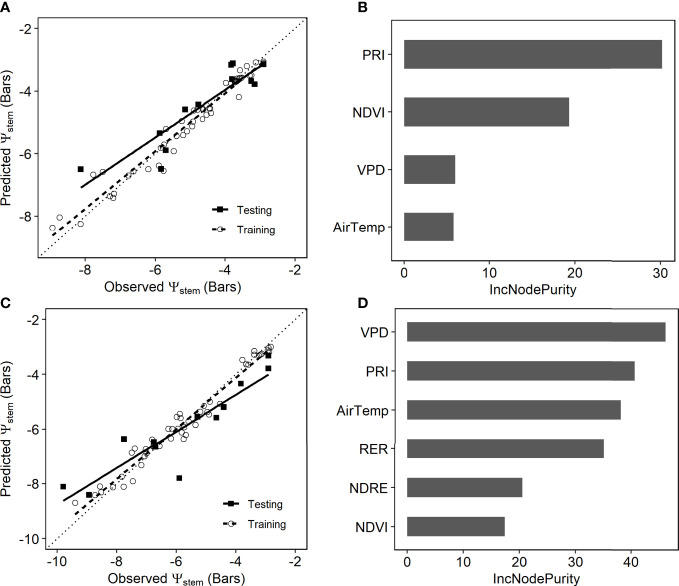
**(A)** Estimated stem water potential (*ψ*_stem_) by the random forest model with the proximal sensor observations compared with ground measurements. Ground measurements from multiple dates were randomly split into training (open circles, dashed line) and testing (solid squares, solid line). **(B)** Variable importance plot of the proximal sensor-based random forest model. **(C)** Estimated stem water potential (*ψ*_stem_) by the unmanned aerial vehicle (UAV)-based random forest model compared with ground measurements. Ground measurements from multiple dates were randomly split into training (open circles, dashed line) and testing (solid squares, solid line). **(D)** Variable importance plots of the UAV-based random forest model.

**Figure 8:**
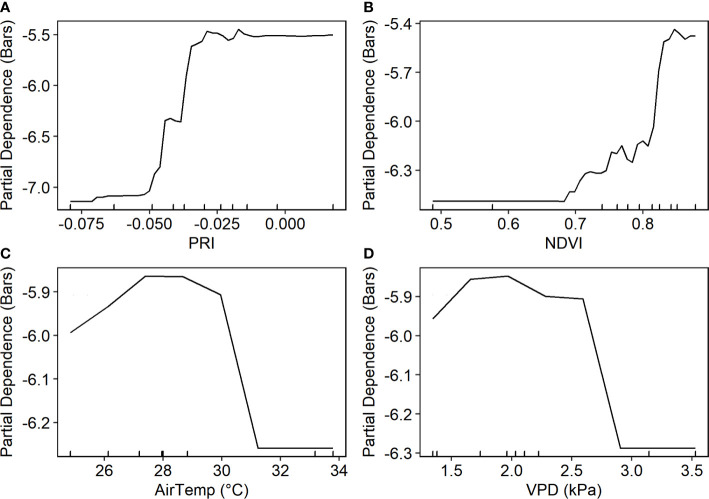
Partial dependence of stem water potential on four predictors based on the proximal sensor-based random forest model.

To better estimate daily mid-day *ψ*_stem_ from proximal sensor-measured PRI and NDVI, VPD and air temperature from weather stations were also added to the random forest model as predictors. The trained RF model explained more than 73% of variance in the testing sets (**Table 3**). The predicted *ψ*_stem_ agreed well with the ground measurements, with RMSE of 0.838 (± 0.123) bars and MAE of 0.626 (± 0.125) bars (**Figure 7A**). PRI was found to be the most important predictor, followed by NDVI (**Figure 7B**). The partial dependence plot showed that *ψ*_stem_ decreased rapidly when PRI increased from -0.05 to -0.025 and when NDVI increased from 0.70 to 0.85 (**Figure 8**). The weather variables VPD and air temperature were less important than the VIs (**Figure 7B**); higher air temperature and VPD decreased *ψ*_stem_ dramatically beyond 28.4°C and 2.0 kPa, respectively (**Figure 8**).

**Table 3:** Random forest model performance in estimating stem water potential (*ψ*_stem_) with proximal sensing data and unmanned aerial vehicle data as represented by the cross-validated mean of the coefficient of determination (*R* ^2^), root mean square error (RMSE), and mean absolute error (MAE) as well as the standard deviation in the parenthesis.

	Model names	Predictors	*R* ^2^	RMSE (bars)	MAE (bars)
Proximal sensor	Full model	PRI, NDVI, AirTemp, VPD	0.737 (± 0.123)	0.838 (± 0.168)	0.626 (± 0.125)
	NoPRI model	NDVI, AirTemp, VPD	0.717 (± 0.129)	0.846 (± 0.158)	0.631 (± 0.138)
UAV	Full model	PRI, NDVI, NDRE, RER, AirTemp, VPD	0.813 (± 0.066)	0.837 (± 0.124)	0.665 (± 0.105)
	NoPRI model	NDVI, NDRE, RER, AirTemp, VPD	0.804 (± 0.072)	0.855 (± 0.132)	0.681 (± 0.106)

Results are shown for full models with four predictors and reduced models excluding weather data and photochemical reflectance index (PRI), respectively.

**Figure 9:**
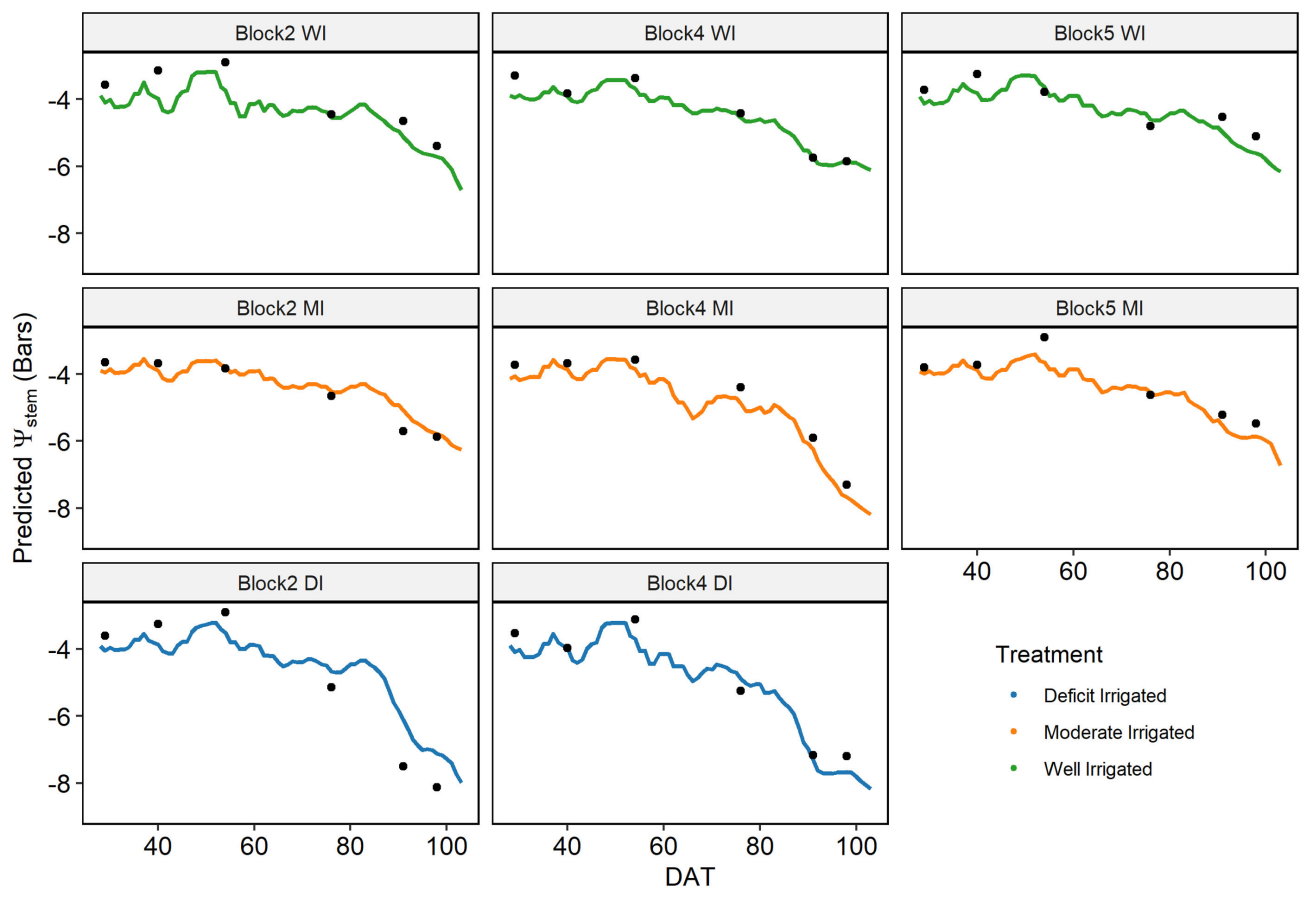
Temporal dynamics of the predicted daily mid-day stem water potential (*ψ*_stem)_ from the proximal sensor-based random forest model during the entire growing season. Field-measured *ψ*_stem_ values are shown as solid dots.

The time series of daily *ψ*_stem_ predicted by the model captured the temporal dynamics during the growing season and across different plots, consistent with field measurements (**Figure 9**). The predicted *ψ*_stem_ was constant approximately -4 bars before June 26, decreased slowly to -6 bars between June 26 and July 17, and then decreased rapidly. The predicted *ψ*_stem_ values of plants under well-irrigated treatments were higher than the plants under moderately irrigated and deficit-irrigated treatments, which agreed well with the spatial pattern of the ground-measured *ψ*_stem._ The predicted *ψ*_stem_ underestimated the *ψ*_stem_ when the *ψ*_stem_ was high (>-4 bars) and overestimated it when the *ψ*_stem_ was very low (<-8 bars).

### 3.3 Relationship between water status and UAV-based VIs

**Figure 10:**
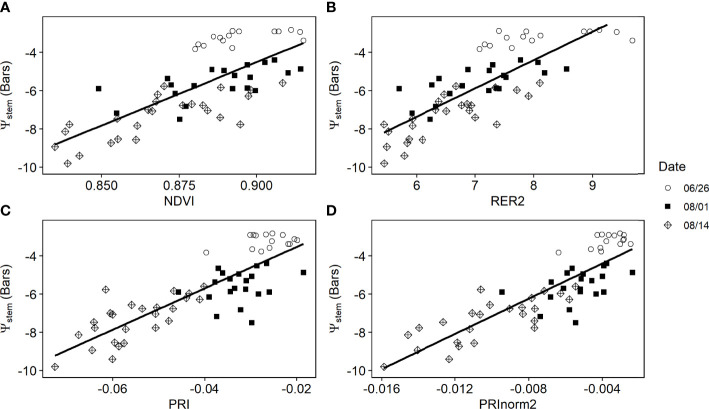
Scatterplots of vegetation indices from the multispectral unmanned aerial vehicle imagery, including **(A)** normalized difference vegetation index, **(B)** Red Edge Ratio 2, **(C)** photochemical reflectance index, and **(D)** normalized photochemical reflectance index *vs*. measured stem water potential (*ψ*_stem_) over the sampled plots.

The majority of VIs derived from UAV imagery had a significant correlation with *ψ*_stem_ (*p*< 0.01) based on the analysis of the concurrent ground measurements and UAV flights over 3 days (*n* = 60) (**Table 2**). Indices related to the xanthophyll cycle, such as PRI and normalized PRI indices, had the strongest linear relationship with *ψ*_stem_, with the correlation coefficient higher than 0.80 (**Figure 10**). VIs representing chlorophyll content, such as RER2 and NDRE1, also correlated well with *ψ*_stem_ correlation coefficient ranging from 0.75 to 0.80. The canopy structure-related VIs had relatively lower correlations with *ψ*_stem_. Among them, NDVI had the highest correlation coefficient (0.55).

When analyzed over each individual day, the correlation between VIs and *ψ*_stem_ showed varying potential of VIs to capture the spatial variability across individual plots. Overall, the correlation coefficient was lower compared with the pooled data, but the correlation increased as *ψ*_stem_ decreased on August 1 and 14 (**Table 2**). The combined indices were significantly correlated with *ψ*_stem_ in all 3 days (*r* > 0.4, *p*< 0.1). Similarly, structural index such as NDVI and some chlorophyll indices such as RER1, RER2, and NDRE1 had a significant correlation with *ψ*_stem_ in all these days. Other VIs only had a high correlation coefficient on August 14 when the plants had lower *ψ*_stem._

### 3.4 Estimation of stem water potential with multispectral UAV imagery

The recursive feature elimination process resulted in six variables for the final full machine learning models, including four VIs (PRI, RER2, NDRE, and NDVI) and two weather variables (air temperature and VPD). The predicted *ψ*_stem_ by the RF model matched well with the ground-based measurements in the testing dataset (**Figure 7C**). The cross-validation showed that the UAV-based RF model captured more than 81% (± 6.7%) of the total spatial and temporal variation in stem water potentials, with RMSE of 0.84 (± 0.12) bars and MAE of 0.67 (± 0.11) bars (**Table 3**).

**Figure 11:**
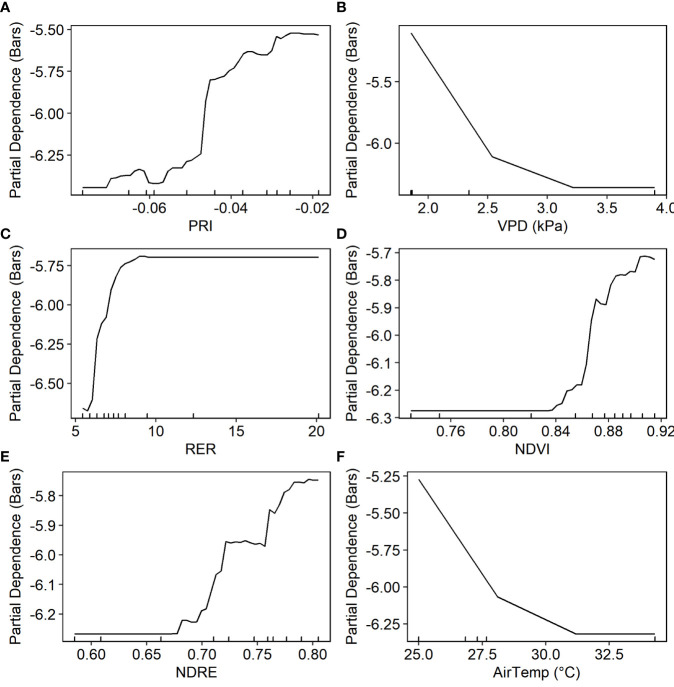
Partial dependence plot of the random forest model based on the data from the unmanned aerial vehicle multispectral imagery and weather station.

VPD and PRI were the top two most important variables, followed by air temperature and RER (**Figure 7D**). The partial dependence analysis showed that higher VPD and air temperature decreased *ψ*_stem_ (**Figure 11**). The *ψ*_stem_ value decreased by approximately 1 bar, when VPD and air temperature increased from 2.0 to 2.5 kPa and from 25.0 to 31.0°C, respectively. Higher PRI, RER2, NDVI, and NDRE were associated with higher *ψ*_stem_ (**Figure 11**). The *ψ*_stem_ decreased rapidly when PRI increased from -0.05 to -0.04, RER2 from 5 to 10, NDVI from 0.84 to 0.88, and NDRE from 0.67 to 0.75.

### 3.5 Whole-field stem water potential mapping at the plant level

**Figure 12:**
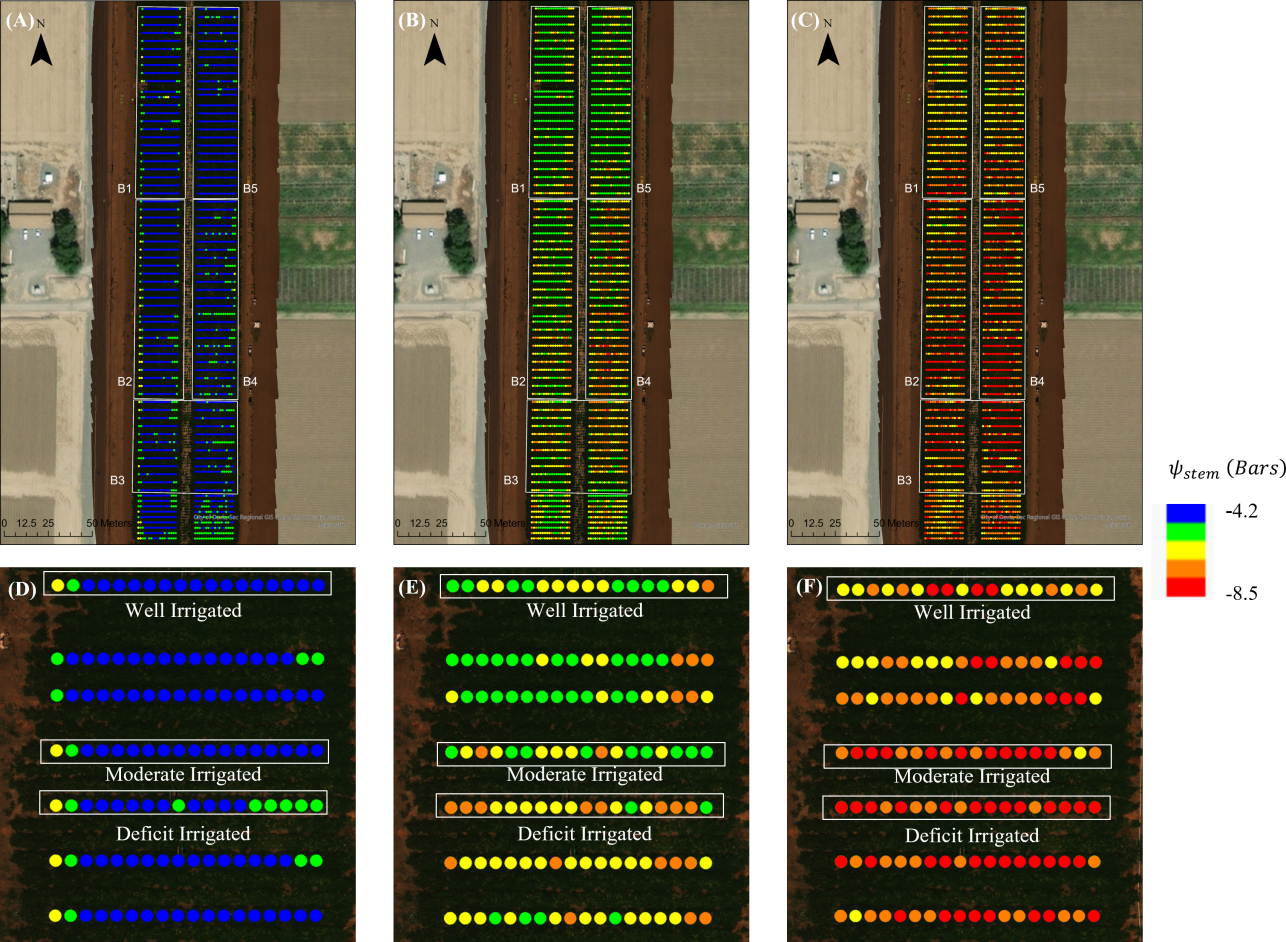
Maps of the plant-level predicted stem water potential (*ψ*_stem_) from the unmanned aerial vehicle-based random forest model for the tomato fields during three dates: **(A)** June 26, **(B)** Aug 1, and **(C)** Aug 14. Also shown is the detailed plant-level predicted *ψ*_stem_ of a sample region in block 2 for the three dates: **(D)** June 26, **(E)** Aug 1, and **(F)** Aug 14.

For those 3 days where UAV imagery was available, we estimated *ψ*_stem_ for every single tomato plant using the RF full model that we developed (**Figure 12**). The maps of the estimated *ψ*_stem_ showed a large spatial heterogeneity within the field, with a coefficient of variation (CV) ranging from 14% to 17% on 3 days. Block 1 and block 5, which were located on the north side of the field, generally had higher *ψ*_stem_ than the other blocks, in all three days, probably due to the different soil properties of the field. The plots under Deficit Irrigated treatment had significantly lower *ψ*_stem_ than plants under other treatments on August 1^st^ (-6.0 ± 0.8 bars) and August 14 (-7.7 ± 0.9 bars). The Well Irrigated plots had the highest *ψ*_stem_ than the other plots in August 1 (-5.5 ± 0.7 bars) and August 14 (-6.7 ± 0.9 bars). The within-plot variability was also visible from the map. On June 26, the plants on the side of the plots had lower *ψ*_stem_ than the plants in the center of the plots. This pattern was less prominent on Aug 1 and Aug 14, as different irrigation treatments contributed more to the spatial variability of plant water status in the late season.

**Figure 13:**
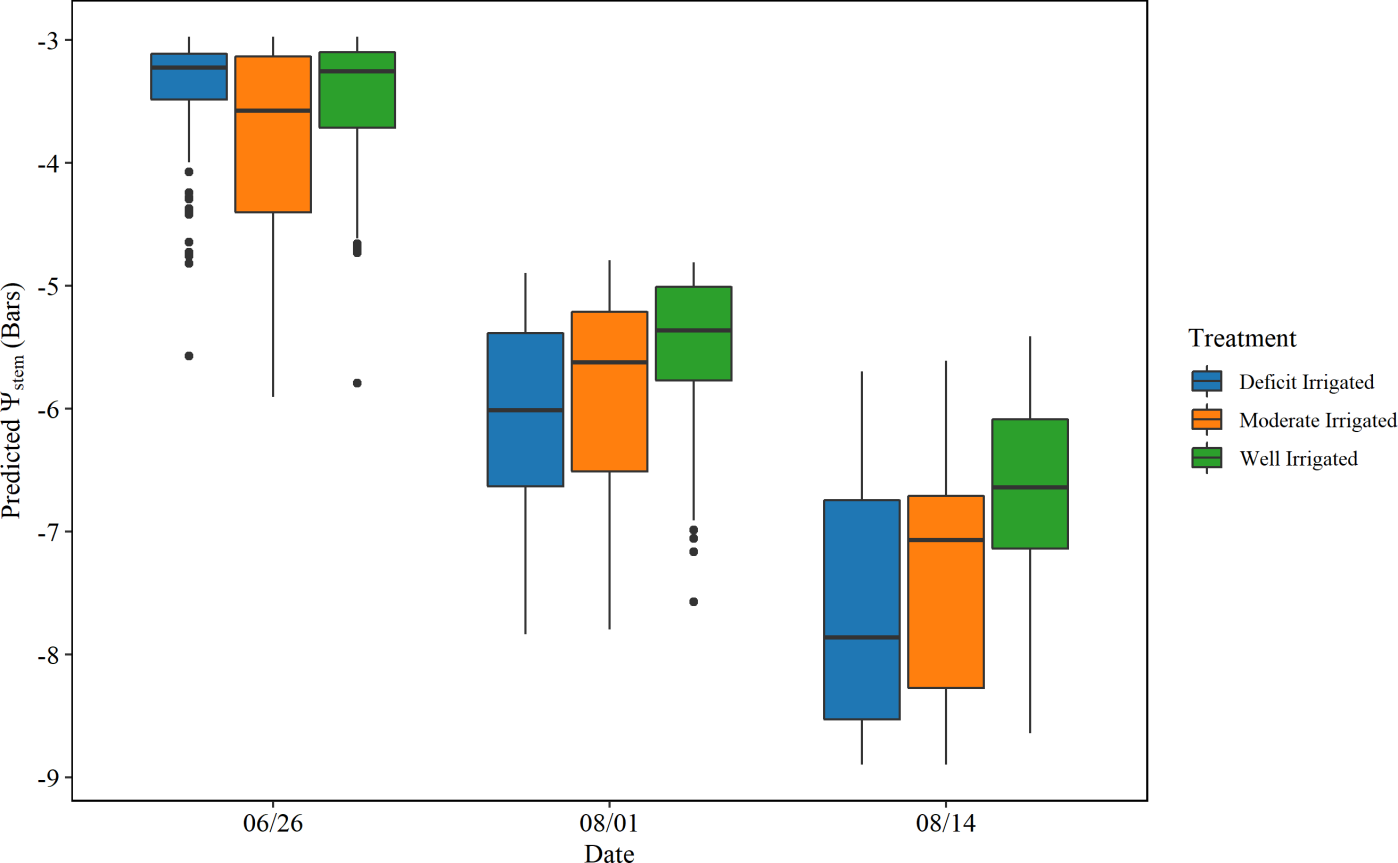
Statistics of the predicted stem water potential (*ψ*_stem_) from unmanned aerial vehicle-based random forest model summarized over plants with different treatments. Each boxplot shows the minimum, first quartile, medium, third quartile, and maximum values *ψ*_stem_.

The water status maps from UAV imagery also captured the day-to-day changes of *ψ*_stem._ A significant decrease of *ψ*_stem_ was found from June 26 to Aug 14 across the entire field and over plots with different treatments (**Figure 13**). The whole field *ψ*_stem_ decreased from 3.5 ± 0.6 bars in June 26 and -5.7 ± 0.8 bars on August 1 to -7.2 ± 1.0 bars in August 14 (**Figure 13**). The *ψ*_stem_ progressed more significantly for plants with deficit irrigation, *i*.*e*., with medium values dropping to -7.9 bars at the end of the growing season. This temporal trajectory was consistent with the field measurements of *ψ*_stem_ and the predicted seasonal change of *ψ*_stem_ from the proximal sensor-based model. In addition, we also found a larger spatial variability of water status after deficit irrigation was applied—for example, the standard deviation across the entire field increased from 0.64 bars in June 26 to 0.97 bars in Aug 14; similarly, the interquartile range (IQR) increased from 0.95 bars to 1.61 bars from June 26 to Aug 14.

## 4 Discussion

### 4.1 Capability of proximal sensing and UAV imaging for water status estimation

Our study demonstrated that machine learning models captured both the spatial and temporal variability of *ψ*_stem_ for processing tomatoes by integrating PRI measurements with other VIs and weather information. Both random forest models, developed separately here for the proximal sensor observations and multispectral UAV imagery, showed comparable or higher accuracy than previous studies on plant water status estimation with similar remote sensing observations (Magney et al., 2016; Saiz-Rubio et al., 2021)—for example, Saiz-Rubio et al. (2021) built a multivariable model with PRI and NDVI from proximal sensors and weather variables to estimate the leaf water potential of grapevines with *R* ^2^ of 0.69 for the testing set (*n* = 36). Another study estimated grapevine *ψ*_stem_ using artificial neural networks to integrate the reflectance at multiple spectral bands including two PRI-related bands and achieved *R* ^2^ values ranging from 0.56 to 0.78 (Poblete et al., 2017).

### 4.2 Impacts of PRI and other VIs on plant water status estimation

The RF model analysis based on both proximal sensors and UAV highlighted that PRI was more important than other VIs in estimating the water status of tomatoes. The univariable linear analysis suggested that PRI signal from proximal sensors can detect the spatial differences of water status on July 17, which was 1 week earlier than NDVI (**Figure 4**). Similarly, in other studies based on proximal sensors (Ihuoma and Madramootoo, 2019) and ground-based spectroradiometer (Alordzinu et al., 2021), it was found that PRI was among the best VIs to distinguish the water status of tomato plants. In addition, PRI has also been shown to be more sensitive than NDVI to water status at the early stages (Rossini et al., 2013). NDVI and other structure-related VIs are generally good indicators of the growth and senescence of green vegetation but often miss or lag short-term responses to plant status that is related with photosynthetic activity (Gamon et al., 2015).

Interpreting PRI signal across the growing season can be challenging due to confounding factors such as canopy structures and chlorophyll and carotenoid absorption (Zarco-Tejada et al., 2013). We found that normalizing PRI with structure and chlorophyll indices can improve the sensitivity to plant water status across time. This is consistent with the findings by Zarco-Tejada et al. (2013) from a grapevine leaf water potential study with multi-spectral airborne imagery.

The chlorophyll-related VIs were also shown to be important for estimating *ψ*_stem_ from UAV imagery. Previous studies have reported that the red edge reflectances are associated with canopy chlorophyll content (Barnes et al., 2000). On one hand, lower chlorophyll content indicates the cumulative impacts of stress on plants, compared with unstressed plants—for example, Ballester et al. (2019) monitored the water stress levels of cotton using RER obtained from UAV imagery, and Eitel et al. (2011) demonstrated the possibility of using NDRE to detect the early stress of conifer woodland. On the other hand, adding red edge information can reduce the confounding factors of PRI and improve the robustness of our model in predicting the plant water status (Zarco-Tejada et al., 2013).

### 4.3 Uncertainties and future work

The remote sensing-based stem water potential monitoring developed in this study provides water status information to guide irrigation management. We recognized that, toward or after the senescence stage, the water status monitoring capability can potentially be confounded, *i*.*e*., due to the slowdown of photosynthetic activities regulated by crop phenology. Previous studies suggest that the canopy-level PRI signal, although influenced by the change of pigment content, can still track the photosynthetic radiation use efficiency at the senescence stage of *Avena sativa* and *Setaria italica*, but the uncertainty could be larger (Cordon et al., 2016).

This study focused on time periods when tomato plants reached full canopy, and therefore the impact of canopy structure and chlorophyll content was reduced in our data and model. During the early stage of plant development, the PRI value is sensitive to changes in canopy cover and LAI, *e*.*g*., when LAI<3 (Barton and North, 2001). Further study is needed to include various growth stages combined with deficit irrigation treatments to fully understand the impacts of other confounding factors on PRI and water status. When the sample size gets bigger, a deep learning approach guided by plant physiology can potentially lead to more explainable and more robust models that can be applied to various crop types.

To take advantage of the high temporal frequency of the proximal sensors, another important step is to explore the potential of a delta PRI based on the difference between the midday PRI and early morning PRI for tracking the plant water stress (Magney et al., 2016). Data fusion approaches can also be applied to make use of the high-temporal-frequency proximal sensors and large coverage of UAV imagery, when both are available, for improved agriculture management (De Benedetto et al., 2013; Schirrmann et al., 2017; Castrignanò et al., 2021).

When the sample size gets bigger, deep learning and machine learning approach guided by plant physiology can potentially lead to more explainable and more robust models that can be applied to various crop types.

### 4.4 Potential application for monitoring crop water status and irrigation management

By combining PRI and other multispectral data from proximal sensors and UAV imagery and weather information with a machine learning model, we can estimate the tomato water status across the entire field. This approach can potentially supplement the traditional ground measurements of *ψ*_stem_ over sampled plants with pressure chambers and scale up to capture the within-field spatial heterogeneity in a timely and efficient way. With the daily change of water status derived from the proximal sensors, growers can track the temporal pattern of irrigation requirements on a daily basis and manage their irrigation schedule more precisely. The water status map produced from UAV imagery can also help the growers to design the irrigation zones and apply informed variable rate irrigation strategies to save water.

## 5 Conclusion

We developed and tested the capabilities of proximal sensing and multispectral UAV imagery for monitoring the water status in a processing tomato field. We found that the proximal sensor-based RF model, driven by PRI, NDVI, VPD, and air temperature, can successfully track the daily change of *ψ*_stem,_ with *R* ^2^ of 0.74 and MAE of 0.63 bars. By integrating multiple VIs from the 12-band UAV imagery and weather, the RF model captured the spatial variability in *ψ*_stem_ at the plant level (*R* ^2^ = 0.81and MAE = 0.67 bars). The xanthophyll index, PRI, was found to be the most important remote sensing variable in both models, providing critical information to capture the spatial and temporal variability of *ψ*_stem._ Our results demonstrated the potential of using PRI and other VIs from proximal sensors and UAV imagery to monitor the plant water status and thus contribute to data-driven irrigation management.

## Data availability statement

The raw data supporting the conclusions of this article will be made available by the authors, without undue reservation.

## Author contributions

ZT: methodology development, data collection and processing, analysis of results, and writing—draft preparation. MP: project director, funding acquisition, methodology development, plot design, data collection and processing, writing—review and editing. YJ: supervision, ideas and analysis, writing—review, and significant editing. PB: funding acquisition, plot design, supervision, writing—review, and editing. All authors contributed to the article and approved the submitted version.
